# Impact of Porphyrin Binding to GENOMES UNCOUPLED 4 on Tetrapyrrole Biosynthesis *in planta*

**DOI:** 10.3389/fpls.2022.850504

**Published:** 2022-03-15

**Authors:** Vincent Fölsche, Christopher Großmann, Andreas S. Richter

**Affiliations:** ^1^Physiology of Plant Cell Organelles, Humboldt-Universität Berlin, Berlin, Germany; ^2^Department of Plant Physiology, Humboldt-Universität Berlin, Berlin, Germany; ^3^Physiology of Plant Metabolism, University of Rostock, Rostock, Germany

**Keywords:** GUN4, porphyrin, tetrapyrrole biosynthesis, post-translation, regulation

## Abstract

Plant tetrapyrrole biosynthesis (TPS) provides the indispensable chlorophyll (Chl) and heme molecules in photosynthetic organisms. Post-translational mechanisms control the enzymes to ensure a balanced flow of intermediates in the pathway and synthesis of appropriate amounts of both endproducts. One of the critical regulators of TPS is GENOMES UNCOUPLED 4 (GUN4). GUN4 interacts with magnesium chelatase (MgCh), and its binding of the catalytic substrate and product of the MgCh reaction stimulates the insertion of Mg^2+^ into protoporphyrin IX. Despite numerous *in vitro* studies, knowledge about the *in vivo* function of the GUN4:porphyrin interaction for the whole TPS pathway, particularly in plants, is still limited. To address this, we focused on two highly conserved amino acids crucial for porphyrin-binding to GUN4 and analyzed GUN4-F191A, R211A, and R211E substitution mutants *in vitro* and *in vivo*. Our analysis confirmed the importance of these amino acids for porphyrin-binding and the stimulation of plant MgCh by GUN4 *in vitro*. Expression of porphyrin-binding deficient F191A, R211A, and R211E in the *Arabidopsis gun4-2* knockout mutant background revealed that, unlike in cyanobacteria and green algae, GUN4:porphyrin interactions did not affect the stability of GUN4 or other *Arabidopsis* TPS pathway enzymes *in vivo*. In addition, although they shared diminished porphyrin-binding and MgCh activation *in vitro*, expression of the different GUN4 mutants in *gun4-2* had divergent effects on the TPS and the accumulation of Chl and Chl-binding proteins. For instance, expression of *R211E*, but not *R211A*, induced a substantial decrease of ALA synthesis rate, lower TPS intermediate and Chl level, and strongly impaired accumulation of photosynthetic complexes compared to wild-type plants. Furthermore, the presence of R211E led to significant growth retardation and paler leaves compared to GUN4 knockdown mutants, indicating that the exchange of R211 to glutamate compromised TPS and Chl accumulation more substantially than the almost complete lack of GUN4. Extensive *in vivo* analysis of GUN4 point mutants suggested that F191 and R211 might also play a role beyond porphyrin-binding.

## Introduction

In photosynthetic organisms, tetrapyrrole biosynthesis (TPS) provides the essential molecules chlorophyll (Chl), heme, siroheme, and phytochromobilin. Chl serves in photosynthesis, heme in electron transfer reactions, siroheme in nitrogen and sulfur assimilation and phytochromobilin in light-regulated gene expression (Tanaka and Tanaka, 2007; Tanaka et al., 2011; Brzezowski et al., 2015). TPS is located in plastids and initiated with the synthesis of 5-aminolevulinic acid (ALA), the common precursor for all tetrapyrroles. In higher plants, algae, and most bacteria, ALA is synthesized from glutamate ligated to tRNA (Glu), which is converted to glutamate 1-semialdehyde (GSA) by GLUTAMYL-tRNA REDUCTASES 1 and 2 (GluTR is encoded by *HEMA1* and *2*). Subsequently, GLUTAMATE 1-SEMIALDEHYDE AMINOTRANSFERASE (GSAAT) catalyzes the formation of ALA. Eight ALA molecules are converted to protoporphyrin IX (PIX), the last common macrocyclic precursor for heme and chlorophyll biosynthesis (Tanaka and Tanaka, 2007; Tanaka et al., 2011; Brzezowski et al., 2015). Within the heme branch of TPS, FERROCHELATASE (FC) inserts Fe^2+^ into PIX, yielding protoheme. Within the Chl branch, ATP-dependent insertion of Mg^2+^ into PIX is catalyzed by MAGNESIUM CHELATASE (MgCh). The MgCh is a multi-enzyme complex composed of the catalytic H subunit (*CHLH*) and two rings of hexameric D and I subunits, respectively (encoded by *CHLI* and *CHLD*) (Lundqvist et al., 2010; Chen et al., 2015a; Adams et al., 2020; Gao et al., 2020). In the two subsequent reactions, Mg protoporphyrin IX (MgP), the product of the MgCh reaction, is converted to protochlorophyllide (Pchlide) by MgP methyltransferase (MgPMT/*CHLM*) and aerobic cyclase (Tanaka and Tanaka, 2007; Tanaka et al., 2011; Brzezowski et al., 2015). In angiosperms, the conversion of Pchlide to chlorophyllide (Chlide) is catalyzed by the strictly light-dependent PROTOCHLOROPHYLLIDE OXIDOREDUCTASE (POR). Subsequently, Chl synthase esterifies Chlide and phytyl-pyrophosphate, and CHLOROPHYLL A OXYGENASE (CAO) catalyzes Chl b formation from Chlide a or Chl a. Chl a and Chl b are then integrated into Chl-binding proteins of the core complexes of photosystem I and II and their outer antenna complexes consisting of light-harvesting chlorophyll-binding proteins (LH) (Mochizuki et al., 2010; Wang and Grimm, 2021).

When unbound Chl and Chl precursors of the TPS pathway absorb light, they can transfer the excitation energy to molecular oxygen and generate highly reactive singlet oxygen (^1^O_2_). Control of the balanced flow of intermediates toward the TPS end-products prevents photooxidative stress by post-translational mechanisms regulating stability and activity of the TPS enzymes and their protein-protein interactions (Moulin and Smith, 2005; Richter and Grimm, 2013, 2019; Brzezowski et al., 2015; Wittmann et al., 2021). The GENOMES UNCOUPLED 4 (GUN4) protein, identified in a screen for retrograde signaling mutants (Susek et al., 1993; Larkin et al., 2003), is a post-translational regulator of Chl biosynthesis in cyanobacteria, green algae and plants (Larkin et al., 2003; Wilde et al., 2004; Sobotka et al., 2008; Peter and Grimm, 2009; Formighieri et al., 2012; Brzezowski et al., 2014). In *Arabidopsis* GUN4 is not essential for basal MgCh activity and Chl formation in continuous low light (Larkin et al., 2003; Peter and Grimm, 2009). However, although details on the molecular mechanism are still missing, GUN4 interaction with the H subunit of MgCh stimulated the chelation of PIX to MgP (Larkin et al., 2003; Davison et al., 2005; Verdecia et al., 2005). In angiosperms, the C-terminus of GUN4 (Zhou et al., 2012; Richter et al., 2016) is crucial, as phosphorylation of the penultimate amino acid controls the stimulation of Mg chelation *in vivo* and *in vitro* (Richter et al., 2016). Also, physical interaction with BALANCE of CHLOROPHYLL METABOLISM1 (BCM1), a thylakoid bound scaffold protein, is essential for the maximum stimulation of MgCh by GUN4 (Wang et al., 2020). Recently it has also been shown that GUN4 is stabilized and prevented from aggregation through the interaction with the chloroplast SIGNAL-RECOGNITION PARTICLE COMPONENT 43 (cpSRP43), thereby promoting high MgCh activity even during heat stress (Ji et al., 2021). In addition to MgCh stimulation, GUN4 was also proposed to regulate ALA synthesis by a yet unknown mechanism (Peter and Grimm, 2009). While overexpression of *GUN4* led to increased ALA synthesis capacity in tobacco, *gun4-3* knockdown mutants showed reduced ALA synthesis. Furthermore, Chl deficiency in response to GUN4-knockout can be partially complemented by exogenous ALA, indicating that diminished ALA synthesis contributes to the Chl deficiency in *gun4-2* (Peter and Grimm, 2009).

**FIGURE 1:**
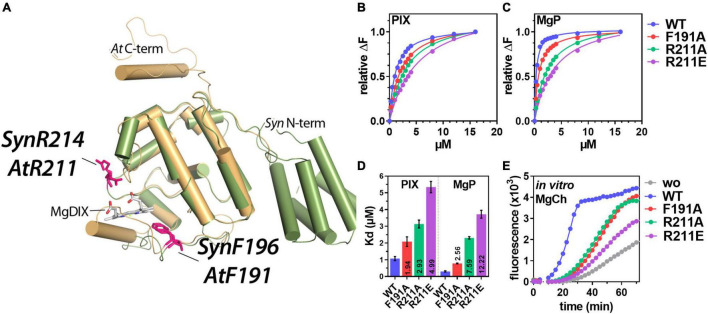
*In vivo* analysis of porphyrin binding and Mg-chelatase (MgCh) activity in the presence of WT and mutant GENOMES UNCOUPLED 4 (GUN4). **(A)** Overlay of the three-dimensional structure of *Synechocystis* (*Syn*) GUN4 (green) and a predicted structure (PHYRE^2^, Kelley et al., 2015) of *Arabidopsis thaliana* (*At*) GUN4 (beige). The crystal of *Synechocystis* sp. PCC 6803 GUN4 (1Y6I) contained the porphyrin derivate - magnesium deuteroporphyrin IX (MgDIX) - which resembles the native product of the MgCh reaction (MgP). Amino acids analyzed in this study are highlighted (pink). **(B,C)** Analysis of PIX **(B)** and MgP **(C)** binding to GUN4(WT) and substitution mutants of GUN4 using Trp-fluorescence quenching analysis. Data points for the relative fluorescence quenching (αF) reflect the mean of a total of five titration curves from two independent protein purifications. The maximum fluorescence quenching efficiency of the highest porphyrin concentration was set to 1. **(D)** Apparent dissociation constants (Kd) for PIX and MgP binding to GUN4, calculated from the binding curves shown in panels **(B,C)** (mean ± S.E.) for *n* = 4 binding curves analyzed with independent protein preparations). Values inside the bars indicate the fold change of the Kd relative to the WT protein. **(E)** *In vitro* MgCh assay using recombinant rice (*Oryza sativa*) MgCh subunits in the absence (gray) or presence of GUN4 (WT) (blue), GUN4(F191A) (red), GUN4(R211A) (green) and GUN4 (R211E) (purple), respectively. The formation of the MgCh reaction (PIX) product was monitored as an increase in fluorescence emission at 595 nm. MgCh assays were repeated three times with proteins from independent purifications, and one representative result is shown.

Biochemical and structural analysis revealed that GUN4 from cyanobacteria, green algae and plants binds the substrate and product of the MgCh reaction but also other TPS intermediates (Davison et al., 2005; Verdecia et al., 2005; Adhikari et al., 2009; Chen et al., 2015b; Tarahi Tabrizi et al., 2015; Hu et al., 2021; Zhang et al., 2021). Tetrapyrrole binding is stabilized by hydrophobic and electrostatic interactions between the substrate(s) and conserved amino acids in helix α2 and a flexible loop between helices α6 and α7 of GUN4 (see also Figure 1). Both structural elements are part of the GUN4 porphyrin binding pocket (Davison et al., 2005; Verdecia et al., 2005; Chen et al., 2015b). MgP binding to *Synechocystis* (*Syn*) GUN4 was shown to be crucial for the stimulatory effect on the MgCh reaction (Verdecia et al., 2005). Based on the approximately 10-fold higher affinity toward MgP than PIX, a function of GUN4 for product release from the MgCh and channeling to the subsequent enzyme MgPMT was proposed. However, altered porphyrin binding to *Thermosynechococcus elongatus* (*Te*) GUN4 did not abrogate MgCh stimulation. Also, *Te*GUN4 increased the sensitivity of MgCh for Mg^2+^ (Davison et al., 2005). Porphyrin-dependent association of pea and *Arabidopsis* GUN4 to thylakoid membranes and the MgCh H subunit was shown (Adhikari et al., 2009, 2011), but this seems to be not the case for *Synechocystis* GUN4 (Kopecna et al., 2015). Despite its impact on MgCh, the binding of porphyrins to GUN4 influences the abundance of GUN4 in high light-treated *Arabidopsis* plants (Adhikari et al., 2011), the stability of MgCH H1 subunit (Zhang et al., 2021) and ^1^O_2_ formation and retrograde signaling in *Chlamydomonas* (Brzezowski et al., 2014; Tarahi Tabrizi et al., 2016).

Although the intensive research on GUN4 provided valuable insights on its function for MgCh and intracellular communication, a comprehensive study on the impact of GUN4:porphyrin complexes on the whole TPS in plants is still missing. Therefore, we complemented the *Arabidopsis gun4-2* knockout mutant with genomic constructs encoding for GUN4 variants with altered porphyrin-binding affinity and analyzed ALA synthesis, steady-state levels of TPS intermediates and end products, the gene expression and the content of proteins in TPS and photosynthetic complexes.

## Materials and Methods

### Plant Material, Growth Conditions, and 5-Aminolevulinic Acid Feeding

If not otherwise stated *Arabidopsis thaliana* wild-type and mutant plants were grown on soil for 14–21 days at 100 μmol photons m^–2^ s^–1^ in short-day (SD) conditions (10 h light). The following genotypes were used in this study: *gun4-1* (EMS mutant, Larkin et al., 2003), *gun4-2* (SALK_0264911, Larkin et al., 2003, maintained in the heterozygous state), *gun4-3* (SALK_011461,Peter and Grimm, 2009), *cao* (SAIL_1238_D01), knockout mutant for *cpSRP43* (*chaos* in Landsberg-0 ecotype), for *cpSRP54* (*ffc*, WiscDsLox289_292B14) and the *chaos/ffc* double mutant (Wang et al., 2018). For long-term ALA feeding, seeds were surface sterilized using ethanol and plated on 0.5 × MS plates (4.4 g/l MS, 0.5 g/l MES, 0.8% Agar, pH 5.7) without or supplemented with 100 or 250 μM ALA, respectively. For ALA feeding in the presence of an exogenous carbon source, 1% sucrose was added to the medium. Plates were kept at 60 μmol photons m^–2^ s^–1^ (continuous light). For short-term ALA feeding, plants were incubated in buffer (20 mM TRIS, pH 7.5) without or supplemented with 1 mM ALA for 20 h in darkness. After incubation, leaves were dried on a tissue and frozen in liquid nitrogen.

### Cloning, Complementation, and Recombinant Protein Expression

Cloning of the complementation constructs encoding *GUN4-F191A*, *-R211A*, *and -R211E* was essentially performed as previously reported (Richter et al., 2016). Genomic DNA fragments were amplified from Col-0 genomic DNA (gDNA) with 800 bp upstream (5′, including the promoter) and 600 bp downstream (3′) of the GUN4 exon (AT3G59400). The following primer were used: CCCGGGCGAAGAATCACCACAATCTAC (fwd, *Sma*I) and CACGTGCCTGTGACGGTTCACACCA (rev, *Pml*I). The GUN4(WT) fragment was cloned into pCambia3301 using *Sma*I and *Pml*I restriction sites. Base exchanges were introduced via a site-directed mutagenesis PCR (Laible and Boonrod, 2009) using the following primers: AtGUN4_F191A_fwd (TTTCCTGACGAAGCCAAGTGGGAGCTT), AtGUN4_F191 A_rev (AAGCTCCCACTTGGCTTCGTCAGGAAA), AtGU N4_R211A_fwd (ACAAACGCCTTGGCAGGAACGCAGCTT), AtGUN4_R211A_rev (AAGCTGCGTTCCTGCCAAGGCGTTT GT), AtGUN4_R211E_fwd (ACAAACGCCTTGGAAGGAACG CAGCTT), AtGUN4_R211E_rev (AAGCTGCGT TCCTTCCAAGGCGTTTGT). The pCambia_GUN4(WT) (Richter et al., 2016) vector was used as template, and base exchange was verified by sequencing. Constructs for recombinant expression of 6xHIS-tagged protein were constructed using the SDM method and primer listed above and the previously published pQE80_AtGUN4(WT) vector as template. Proteins were expressed and purified as described before (Richter et al., 2016). Complementation of the heterozygous *gun4-2* mutant, selection and genotyping of homozygous transformants was performed as previously reported (Richter et al., 2016).

### Porphyrin, Pigment, and Heme Analysis

Chlorophyll, TPS intermediates and heme were extracted from 50 to 100 mg of ground and lyophilized plant material using 1 mL acetone:0.2 M NH_4_OH (9:1, v/v) at −20°C for at least 30 min. Samples were centrifuged for 10 min at 4°C (14,000 g), supernatants were transferred to new reaction tubes and centrifuged again for 30 min at 4°C (14,000 g). The remaining pellets of Chl extraction were used to extract non-covalently bound heme with 200 μL acetone:HCl:DMSO; 10:0.5:2 (v/v/v) for 20 min at RT. Following centrifugation (30 min, 14,000 g, RT) supernatants were subjected to HPLC analysis. HPLC analysis for pigments, heme and intermediates was performed on Agilent HPLC systems, essentially as described previously (Schlicke et al., 2014; Scharfenberg et al., 2015).

### RNA, cDNA, and qPCR

RNA was extracted from frozen leaf tissue following the protocol published by Oñate-Sánchez and Vicente-Carbajosa (2008). 1–2 μg DNase treated RNA (ThermoFisher Scientific, MA, United States), were transcribed to cDNA using the Revert Aid RT (ThermoFisher Scientific, MA, United States) and oligo dT(18) primer following the manufacturer protocol. qPCR analysis was carried out using a Bio-Rad CFX Connect Real-Time PCR Detection System and SYBR green qPCR master mix (ChamQ Universal SYBR^®^ qPCR Master Mix, Absource, ger). Primer for gene expression analysis were published before (Richter et al., 2013, 2016), and expression was normalized to *SAND* (AT2G28390) and calculated relative to the WT samples using the 2^–ΔΔC(t)^ method.

### Protein Extraction and Western-Blot Analysis

Frozen plant material was homogenized and proteins were extracted with protein extraction buffer [PEB; 2% (w/v) SDS, 56 mM Na_2_CO_3_, 12% (w/v) sucrose, 56 mM DTT, and 2 mM EDTA, pH 8.0] using a fixed ratio of 10 μl PEB per mg of fresh weight. After homogenization, samples were incubated for 20 min at 70°C and subsequently centrifuged for 10 min at 14,000 g at RT. The protein extracts were stored at −20°C until further use. Proteins were separated using SDS-polyacrylamide gels (SDS-PAGE) and blotted onto nitrocellulose membranes (Amersham Protran, GE Healthcare, United Kingdom) via semidry electroblotting. Membranes were probed with specific antibodies.

### Determination of 5-Aminolevulinic Acid Synthesis Capacity

To determine the ALA-synthesis capacity, leaves were harvested and incubated in buffer supplemented with levulinic acid (20 mM Tris, 40 mM levulinic acid, pH 7.2). After 4–5 h, the plant material was harvested and frozen in liquid nitrogen. The homogenized leaf material was resuspended in 1 mL 20 mM potassium phosphate buffer (pH 6.8). After centrifugation (10 min, 14,000 g, 4°C), supernatants (400 μl) were mixed with 100 μL EAA (ethyl acetoacetate) and boiled for 10 min at 95°C. Then, 500 μL of modified Ehrlich’s reagent [373 ml of acetic acid, 90 ml of 70% (v/v) perchloric acid, 1.55 g of HgCl_2_, 9.10 g of 4-dimethylaminobenzaldehyde, water up to 500 ml] was added to each sample and centrifuged for 5 min (14,000 g at 4°C). The absorption of the ALA pyrrole was measured at 526 nm, 553, and 720 nm using a spectrophotometer. ALA content was calculated based on a standard curve prepared with ALA (Sigma-Aldrich, St. Louis, MO, United States).

### Magnesium Chelatase Assay and Binding Affinities

The binding affinities of *At*GUN4 variants for porphyrins were determined by tryptophane quenching analysis following previously published protocols (Larkin et al., 2003; Richter et al., 2016). AtGUN4 variants (0.5 μM) were incubated in quenching buffer (50 mM Tricine, 300 mM glycerol, 1 mM DTT, pH 7.9) with increasing amounts of porphyrins (10 mM PIX and MgP stocks in DMSO). Tryptophan fluorescence was measured with excitation and emission wavelengths set to 280 and 340 nm, respectively. The apparent dissociation constants (Kd) were calculated using a one site-specific binding modell (GraphPad Prism). *In vitro* MgCh assays were performed following previously published protocols using a fluorescence photometer equipped with a 96-well plate reader (Ex 416 nm/Em595 nm for PIX) and a substrate concentration of 6 μM PIX (Zhou et al., 2012; Richter et al., 2016).

### Thylakoid Extraction and Blue-Native Polyacrylamide Gels

For thylakoid extraction, the leaves of 6–7-week-old plants were harvested and homogenized using a motor and pestle on ice in 14 mL thylakoid extraction buffer (450 mM sorbitol, 20 mM tricine-KOH [pH 8.4], 10 mM EDTA, 0.1% BSA). The extract was filtered through one layer of Miracloth, and the filtrate was centrifuged for 4 min at 4°C and 4,000 rpm. The pellet was resuspended in 14 mL BN shock buffer (50 mM HEPES-KOH [pH 7.5], 5 mM sorbitol, 5 mM MgCl_2_) and centrifuged at 4,000 rpm, 4°C for 4 min. The supernatant was discarded, and the thylakoid pellet was resuspended in 400–600 μL 25BTH20G buffer (25 mM BisTris [pH 7.0], 20% glycerol) and frozen at −80°C. Chlorophyll concentration was determined using the absorption of pigment extracts (see above) at 646 and 663 nm. The concentration of chlorophyll a and b was calculated using the formula Chl a + b (μg/μL) = (17.76 × A_646 *nm*_ × Dilution factor) + (7.34 × A_663 *nm*_ × Dilution factor). Thylakoid proteins were solubilized from an aliquot corresponding to 32 μg Chl using 1% ß-dodecyl maltoside. BN-PAGE was performed according to Peng et al. (2008) on 4–12.5% BN gels (10 μg Chl per sample).

### Chlorophyll Fluorescence Analysis

To determine the maximum Chl fluorescence in the dark-adapted state a saturating light pulse was applied after 20 min dark incubation using a PAM-Imager (MAXI version, WALZ, Germany).

## Results

### Porphyrin Binding and Magnesium Chelatase Activity

Previous analysis of *Synechocystis* (*Syn*) GUN4 and *Arabidopsis* (*At*) GUN4 revealed that the conserved *At*Phe-191 (corresponds to SynPhe-196) and *At*Arg-211 (SynArg-214) (Figure 1A) contribute to the binding of porphyrins to GUN4 (Verdecia et al., 2005; Adhikari et al., 2009, 2011). To analyse the impact of these amino acids on *Arabidopsis* GUN4 function, 6xHIS-tagged WT and point mutants of *Arabidopsis* GUN4 with amino acid substitutions for phenylalanine 191 (F191A) or arginine 211 (R211A and R211E) were heterologously expressed and purified from *E*. *coli* lysates. The latter two substitutions replace arginine with the small and hydrophobic alanine or negatively charged glutamate (Figure 1A). For the analysis of porphyrin-binding affinity, proteins were subjected to tryptophan fluorescence quenching analysis in the presence of an increasing amount of PIX or MgP, respectively (Figures 1B–E). As calculated from the fluorescence quenching curves (Figures 1B,C), all three point mutations compromised the affinity of the mutated GUN4 relative to the WT protein. The WT protein showed apparent dissociation constants (Kd) of 1.05 ± 0.12 and 0.30 ± 0.03 μM for PIX or MgP, respectively (Figure 1D). Relative to GUN4(WT), the F191A mutation led to a 2-fold decrease in the affinity for PIX (Kd = 2.1 ± 0.28 μM) and a 2.5-fold decreased affinity for MgP (Kd = 0.77 ± 0.02 μM; Figure 1D). Compared to F191A, mutation of R211 had a much stronger effect on the porphyrin binding, particularly for MgP. On the one hand, R211A and R211E showed a 3 and 5-fold, respectively, increase in the Kd for PIX, indicating lower affinity compared to the GUN4(WT). On the other hand, the affinity for MgP decreased by 7.5-fold for R211A (Kd = 2.3 ± 0.07 μM) and 12.2-fold for R211E (Kd = 3.2 ± 0.23 μM; Figure 1D).

In order to test the impact of the point mutations on GUN4’s ability to stimulate the MgCh reaction, the purified proteins were subjected to an MgCh-assay containing the recombinant rice subunits CHLH, D and I (Supplementary Figure 1; Zhou et al., 2012). As reported before, the addition of GUN4 (WT) strongly stimulated the MgCh reaction compared to the control reaction without GUN4 (Figure 1E). In contrast, either of the GUN4 point mutants showed diminished stimulation of MgCh and a prolonged lag-phase relative to the reaction with WT GUN4. Despite the overall reduction, we found quantitative differences in the potential to stimulate the MgCh reaction. Both F191A and R211A showed a 50% reduction in the MgCh activity calculated as fluorescence change per minute (ΔF/min) from the slope of the curve (Figure 1E). In contrast, the addition of R211E led to a 70% reduction in MgCh activity (calculated as ΔF/min) and overall limited product formation compared to the GUN4 (WT) reaction.

### Complementation of the *gun4-2 Knock Out* Mutant

**FIGURE 2:**
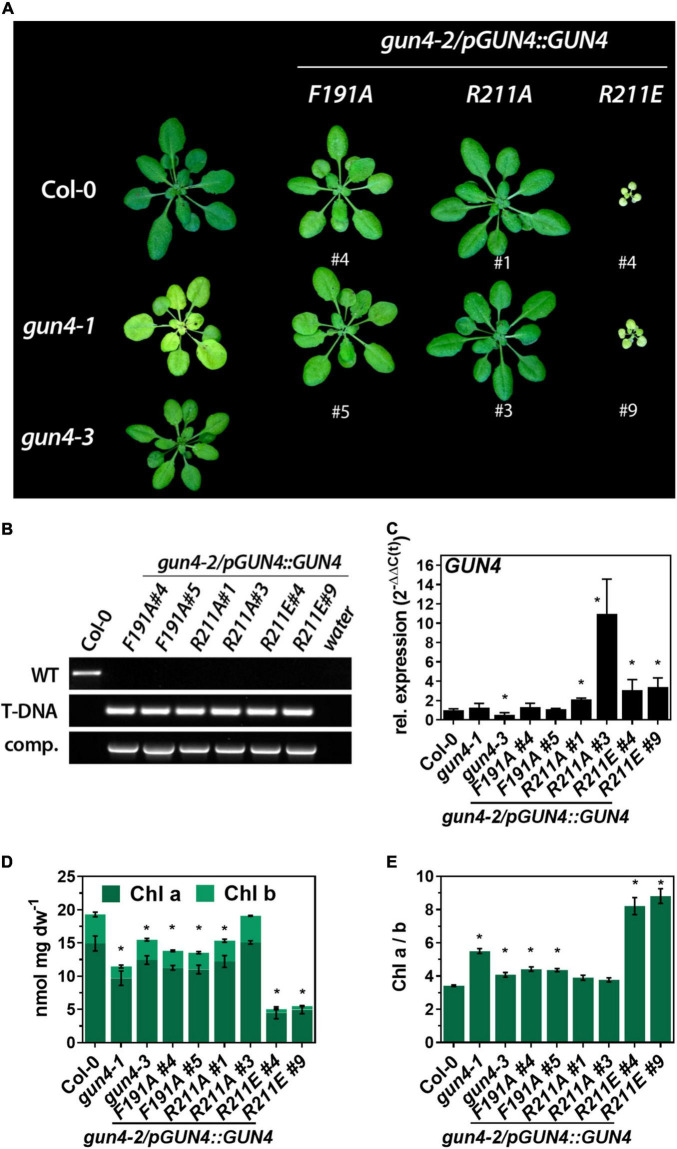
Complementation of the *gun4-2* knock out mutant with genomic constructs encoding for *GUN4*(*F191A*), *GUN4*(*R211A*), and *GUN4*(*R211E*). **(A)** Phenotype of 5-week-old WT (Col-0) and mutant plants grown on soil under short-day conditions at 100 μmol photons^– 2^ s^– 1^. In addition, *gun4-1* and *gun4-3* knock out plants are shown. **(B)** PCR-based confirmation of the *gun4-2* knock out background and presence of the complementation construct in the mutant lines shown in panel **(A)** using primers for the amplification of the *GUN4* WT-allele (WT), the SALK_026911 T-DNA insertion (T-DNA) and the complementation construct (comp.) **(C)** Expression of *GUN4* mRNA relative to Col-0 and SAND as reference gene [2^–ddC(t)^]. **(D)** Chlorophyll (Chl) a and b content and **(E)** Chl a/b ratio. Statistical significance relative to the WT (*p < 0.05) was calculated using one-way ANOVA and Dunnett’s multiple comparison test with *n* ≥ 3 **(C)** and *n* = 4 **(D,E)** replicates. Values are given as the mean ± S.D.

**FIGURE 3:**
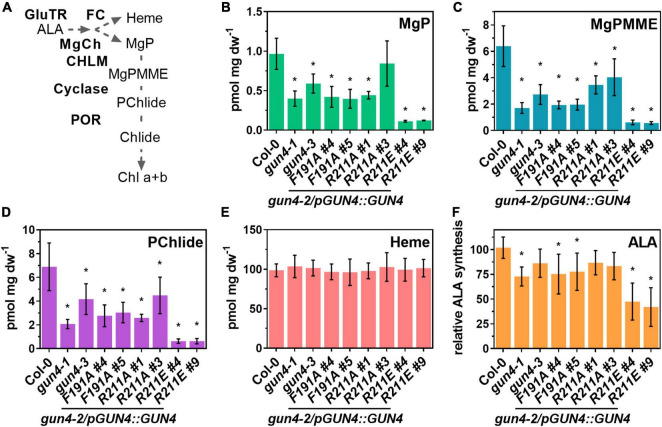
Analysis of porphyrin steady-state level, heme content and ALA synthesis capacity in *gun4-2* complementation lines expressing *GUN4*(*F191A*), *GUN4*(*R211A*), and *GUN4*(*R211E*). **(A)** Schematic presentation of the tetrapyrrole biosynthesis, including the intermediates within the Chl branch and the involved enzymes. Abbreviations are given in the main text. Steady state level of **(B)** Mg-protoporphyin IX (MgP), **(C)** Mg protoporphyin IX monomethylester (MgPMME), **(D)** Protochlodophyllide (PChlide), **(E)** heme, and **(F)** ALA synthesis capacity (relative to the WT control) in leaves of 21 days old plants grown in short-day (SD). Statistical significance relative to the WT (*p < 0.05) was calculated using one-way ANOVA and Dunnett’s multiple comparison test with *n* ≥ 7 (**B–E**, except R211A#1, *n* = 4) and *n* ≥ 7 **(D–F)** replicates from two independent experiments. Values are given as the mean ± S.D.

To test the impact of the GUN4 point mutations on its function *in vivo*, we introduced transgenes encoding mutant versions of *GUN4* under the control of the endogenous GUN4 promoter into the *gun4-2* knockout mutant. All *GUN4* variants used in this study were able to at least partially complement the *gun4-2* knock out mutant, which is seedling lethal in the absence of an exogenous carbon source (Larkin et al., 2003; Figure 2). Two individual homozygous lines per construct were selected for further analysis (Figures 2A,B). Except for the strong overexpressing *R211A* line #3, all complementation lines expressed the transgene at least WT-like (Figures 2C, 3A). In addition to the *gun4-2* complementation lines, we included the *gun4-1* [expressing GUN4(Leu88Phe)] and the T-DNA insertion mutant *gun4-3* to our analysis (Peter and Grimm, 2009). These two allelic knockdown mutants showed a pale green phenotype compared to WT plants, with *gun4-1* being the stronger compromised allele (Figure 2A). While the *gun4-1* mutant accumulated only 60% of WT chlorophyll (Chl) level, *gun4-3* accumulated approximately 80% of Chl found in WT leaves (Figure 2D). Expression of *F191A* led to pale green leaves (Figure 2A) due to diminished Chl accumulation (Figure 2D). This result resembled the phenotype of previously reported independent transgenic lines (Adhikari et al., 2011). In contrast to *F191A*, a different phenotype was observed for the two complementation lines expressing *R211A*. While line #1 accumulated approximately 80% of WT Chl level, line #3 markedly overexpressing *R211A* (Figures 2C, 3A) accumulated WT levels of Chl (Figure 2D). In stark contrast to *R211A*, *R211E* lines were visibly growth-retarded (Figure 2A) and accumulated only 20% of WT Chl level (Figure 2D). Interestingly, the expression of *R211E* compromised the overall Chl accumulation and, in particular, the Chl b content resulting in a drastic change of the Chl a/b ratio compared to the other *gun4* mutant lines (Figure 2E). While *F191A* lines resembled the *gun4* knockdown mutants, expression of the *R211E* variant resulted in the most severe macroscopic phenotype and compromised TPS.

### The Steady-State Level of TPS Intermediates and 5-Aminolevulinic Acid Synthesis Capacity

Because the lower accumulation of Chl in the GUN4 mutant lines depends on the activity of TPS enzymes, primarily MgCh, we analyzed intermediates of the Chl branch of the pathway in WT and mutant plants (Figure 3A). Overall, the steady-state level of porphyrin intermediates confirmed lower Chl accumulation in each line (Figures 3B–D). The steady-state levels of MgP, Mg protoporphyrin IX monomethylester (MgPMME) and Pchlide, which are the products of the catalytic reactions of MgCh, MgPMT and the cyclase, respectively, were significantly reduced in the *gun4-1* and *gun4-3* mutants as well as the *F191A*, *R211A#1*, and *R211E* lines. However, a quantitative difference of these TPS metabolites was detectable among the complementation lines. Expression of *F191A* and *R211A* resulted in a 40–50% decrease in the steady-state levels. In contrast, in the two independent *R211E* lines only 5–10% of WT-levels for MgP, MgPMME, and Pchlide were determined (Figures 3B–D). It is important to note that in all the genotypes, the steady-state level for the substrate of the MgCh, PIX, was below the detection limit when analyzed by HPLC. Despite the strong effect on the Chl branch of the TPS, the content of non-covalently bound heme was not affected in any of the mutants under study (Figure 3E). In order to test to which extent the rate-limiting step of TPS is affected by the different GUN4 mutations, ALA synthesis capacity was analyzed. A mild reduction of ALA synthesis was observed for *gun4-3* and *R211A* lines. Significant differences in the ALA synthesis capacity were detected for the mutants with pale-green leaves and low Chl content (i.e., *gun4-1*, *F191A*, and *R211E* lines) (Figure 2). In particular, in both *R211E* lines, ALA synthesis was diminished by 50–60% compared to Col-0.

### Accumulation of TPS Enzymes and Subunits of Photosynthesis and Gene Expression

**FIGURE 4:**
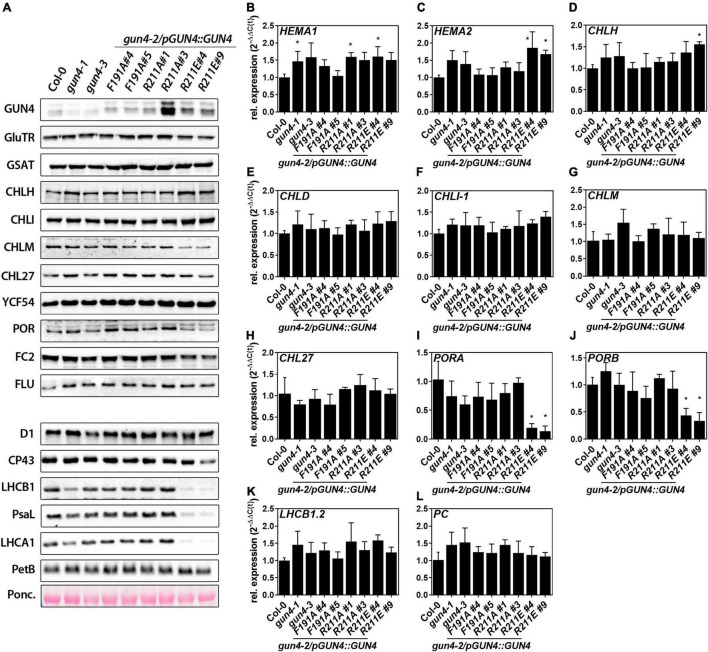
Analysis of protein contents and gene expression in 21 day-old *gun4-2* complementation lines expressing *GUN4*(*F191A*), *GUN4*(*R211A*), and *GUN4*(*R211E*). **(A)** Western-blot analysis of enzymes and regulators involved in tetrapyrrole biosynthesis. **(B–L)** Analysis of mRNA contents by quantitative real-time PCR. Relative expression was calculated relative to Col-0 and *SAND* as reference gene [2^–ddC(t)^]. Statistical significance relative to the WT (*p < 0.05) was calculated using one-way ANOVA and Dunnett’s multiple comparison test with *n* ≥ 3 replicates. Values are given as the mean ± S.D. Plants were grown for 21 days in short-day (SD) conditions. GUN4, Genomes uncoupled 4; GluTR/*HEMA1* and 2, Glutamyl-tRNA reductase; GSAT, Glutamate-1-semialdehyde aminotransferase; CHLH, CHLI(*-1*), CHLD, subunits of the MgCh; CHLM, MgP methyltransferase; CHL27 and YCF54, subunits of cyclase; PORA and B, Protochlorophyllide oxidoreductase; D1, CP43, PsaL, LHCA1 and B1, photosystem I and II subunits; PetB, Cytochrome b6f complex protein; *PC*, plastocyanin.

To analyse if the phenotypic and molecular changes of the substitution mutants (Figures 2, 3) can be explained by transcriptional or post-translational changes, we performed a comprehensive analysis of mRNA expression and the TPS enzyme abundance. Except for the *gun4-1* mutant, *GUN4* expression and the GUN4 protein content correlated in the different genotypes (Figures 2C, 4A). The key enzymes involved in ALA synthesis, GluTR (*HEMA1* and *2*) or GSAT, were neither affected on the transcript nor the protein level (Figures 4A–C). Transcripts for the MgCh subunits *CHLH*, *D*, and *I-1* accumulated at WT-level in all mutants (Figures 4D–F). However, and in agreement with previous results (Wang et al., 2020), CHLH was more abundant in *gun4-1* and also in the *gun4-2/R211E* lines. Although a reduction for CHLM and POR protein was observed, only the transcripts for *PORA* and *B* were strongly diminished in the *R211E* lines (Figures 4I,J). Furthermore, no changes in the accumulation of *LHCB1*.*2* and plastocyanin (*PC*) mRNA were found in fully developed leaves of the genotypes analyzed here (Figures 4K,L). In addition to the TPS enzymes, the abundance of photosynthetic complex proteins was also analyzed (Figure 4A). Except for *R211E#9*, only a minor difference in the accumulation of photosystem (PS) II subunits D1 and CP43 was observed. Accumulation of the PSI subunit (PsaL) was impaired in *gun4-1* and to a stronger extent in *R211E* lines. Likewise, the accumulation of LHC antenna proteins (LHCB1 and LHCA1) was diminished in *gun4-1*, *gun4-3*, the *F191A* lines and *R211A#1* compared to Col-0 (Figure 4A). In *R211E* LHCA1 and B1 were barely detectable and accumulated to a lower level compared to the *gun4-1* knockdown mutant (Figure 4A). The cytochrome b(6) subunit of the cytochrome b6f complex (PetB) was detected with similar amounts in all lines tested.

### Effect of Short- and Long-Term 5-Aminolevulinic Acid Feeding on Porphyrin and Chlorophyll Accumulation

**FIGURE 5:**
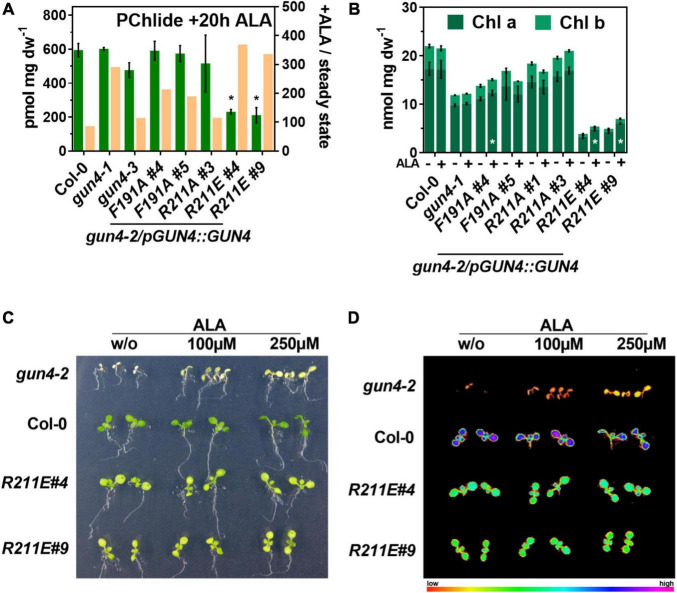
Impact of short and long-term ALA feeding on protochlorophyllide (PChlide) and chlorophyll (Chl) accumulation in *gun4* mutants. **(A)** PChlide content of 21 days old seedlings grown in short-day incubated in buffer containing 1 mM ALA for 20 h in darkness (green). The ocre bars indicate the change in PChlide content upon ALA feeding relative to the steady-state level in the light (ratio + ALA/steady-state, right Y-axis). **(B)** Chl a and b content in WT and mutant plants germinated and grown for 14 days in continuous light on MS plates without (–) or supplemented with 250 μM ALA (+). Statistical significance (*p < 0.05) relative to the WT (in A) or the control condition without ALA (in B) was calculated using student’s t-test with *n* = 3 replicates. Values are given as the mean ± S.D. **(C)** Phenotype and **(D)** maximum Chl fluorescence (Fm) induced by a saturating light pulse after 20 min of dark incubation using a PAM-imager. Increased Chl contents in *gun4-2* after ALA feeding are indicated by pale-green cotyledons and higher fluorescence of Chl molecules. Plants were grown on MS media with 1% sucrose without or supplemented with the indicated ALA concentrations in continuous dim-light for 8 days. For *gun4-2*, seeds of a heterozygous plant were sown, and white *GUN4* knockout mutants were selected for the analysis shown.

The strongly diminished TPS observed for some of the complementation lines raised the possibility that a shortage of ALA (Figure 3F) caused the reduced porphyrin and Chl levels and Chl-binding protein contents, particularly in the *R211E* lines. To test this, 21 days old seedlings were incubated in buffer without or supplemented with 1 mM ALA (Figure 5A). To avoid deleterious effects arising from reactive oxygen species (ROS) formation from elevated porphyrin levels during light exposure, plants were incubated for 20 h in darkness. Because POR is strictly light-dependent in angiosperms, we used the accumulation of its substrate, Pchlide, as a proxy for the consumption of exogenous ALA within the TPS in darkness. ALA feeding of WT leaves resulted in high amounts of Pchlide level, which were increased by approximately 110-fold compared to the steady-state level in the light (Figures 4D, 5A). Exogenous supply of ALA fully complemented Pchlide deficiency (Figure 3D) in the knockdown mutants *gun4-1* and *gun4-3*. In particular, for *gun4-1*, a relative increase of 300-fold compared to the light Pchlide content was found. Also, *F191A* lines and the strong overexpressor of *R211A#3* accumulated WT-like levels of Pchlide after ALA feeding (Figure 5A). Despite a relative increase after ALA feeding in darkness by 300–400 fold compared to the content in the light, both *R211E* lines accumulated only 30% of the Pchlide amounts found in the WT after ALA supply (Figure 5A). However, it is noteworthy that none of the lines accumulated the substrate of the MgCh (PIX) or any other intermediate downstream of MgCh (except for Pchlide). In order to test if long-term ALA supply could complement Chl deficiency, plants were grown on MS plates with 250 μM ALA for 14 days in continuous light (Figure 5B). In comparison to control conditions, growth in the presence of ALA did not affect the Chl a and b content in WT, *gun4-1*, *gun4-3* and both *R211A* lines. Although a significant increase of Chl a for *F191A#4* after growth on ALA plates was found, the second line #5 did not show a change in Chl level upon ALA supply. On the contrary, while the Chl b content was not significantly changed, both *R211E* lines accumulated approximately 25% more Chl a after growth on plates with ALA compared to control plates without ALA (Figure 5B). In an independent experiment, WT, the *gun4-2* knockout mutant and *R211E* lines were grown in the absence or presence of increased amounts of ALA on MS plates supplemented with sucrose in continuous dim-light (Figures 5C,D). In agreement with a previous report (Peter and Grimm, 2009), Chl deficiency of the white *gun4-2* mutant can be partially rescued by ALA feeding as indicated by cotyledons becoming pale-green (Figure 5C) and higher maximum Chl fluorescence (Figure 5D) after a saturating light pulse using a PAM-imager. In contrast, ALA supply did not change the low Chl contents and the Fm in the *R211E* lines compared to WT, thereby confirming the results from ALA-feeding in the absence of sucrose.

### Comparison of *R211E* With Other Chlorophyll Deficient Mutants

**FIGURE 6:**
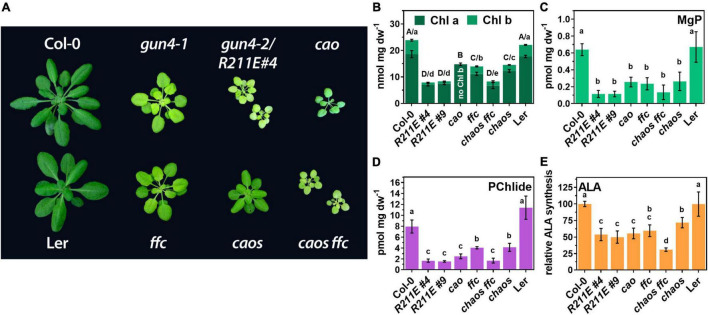
Comparison of *gun4* mutants with mutants impaired in the accumulation of photosynthesis proteins. **(A)** Phenotype of 6-week old WT (Col-0) and mutant plants grown on soil under short-day conditions at 100 μmol photons^– 2^ s^– 1^. In addition to *gun4-1* and the *gun4-2/R211E* mutant, single and double mutants for the chloroplast signal recognition particle (cpSRP) 43 (*chaos*) and cpSRP54 (*ffc*) and the knock out mutant for the chlorophyll a oxygenase (*cao*) are shown. *Chaos* mutant is in Ler background. **(B)** Chl a and b, **(C)** MgP, **(D)** PChlide content, and **(E)** ALA-synthesis capacity in 21 days old plants grown in short-day (SD). Significance groups (*p < 0.05) were calculated using one-way ANOVA and Turkey’s multiple comparison test with *n* ≥ 5 replicates from two independent experiments. Values are given as the mean ± S.D.

**FIGURE 7:**
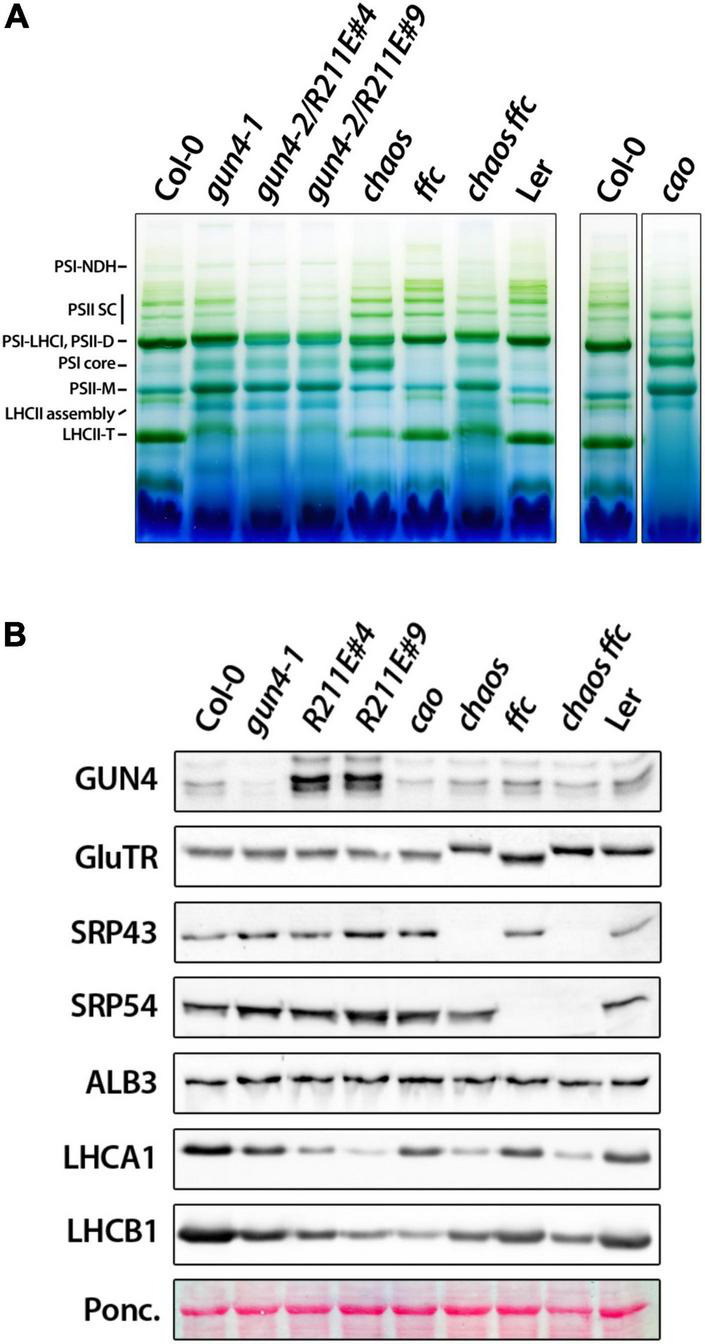
Accumulation of photosynthetic complexes and proteins in *gun4*, chlorophyll b deficient *cao* mutant, and mutants impaired the cpSRP pathway. **(A)** BN-PAGE analysis of photosynthetic complexes solubilized with 1% ß-dodecyl maltoside from thylakoid membranes extracted from 4 to 5 week-old plants grown on soil-grown under short-day conditions at 100 μmol photons^– 2^ s^– 1^. In addition to *gun4-1* and the *gun4-2/R211E* mutant, single and double mutants for the chloroplast signal recognition particle (cpSRP) 43 (*chaos*) and cpSRP54 (*ffc*) pathway and the knockout mutant for the chlorophyll a oxygenase (*cao*) were analyzed. *Chaos* mutant is in Ler background. Complexes were labeled according to Fu et al. (2007). **(B)** Accumulation of proteins involved in tetrapyrrole biosynthesis (GUN4 and GluTR), the cpSRP pathway (SRP43, 54, and ALB3), LHCA1 and B1 analyzed by western-blot analysis of denatured protein extracts obtained from 21 days-old plants grown on soil.

The analysis of the TPS pathway disclosed that *R211E* expression resulted in a rather drastic (molecular) phenotype compared to the knockdown mutant *gun4-1*. We speculated that some aspects of the (molecular) phenotype of *R211E* mutants were caused by effects independent of GUN4s function in MgCh reaction. Therefore, we compared the *R211E* lines with a mutant devoid of Chl b due to CHLOROPHYLL A OXYGENASE (*cao*) deficiency (Figure 6) and mutants for the chloroplast SIGNAL RECOGNITION PARTICLE43 AND 54 complex (*ffc/srp54* and *chaos/srp43*) with impaired integration of Chl-binding proteins into thylakoid membranes (Schuenemann et al., 1998, 1999; Dunschede et al., 2015). The *cao* mutant was similar growth retarded compared to *R211E*. Deficient Chl b biosynthesis resulted in ∼80% of WT Chl a level (Figure 6B) but a minor reduction in leaf pigmentation than *R211E#4* (Figure 6A). The steady-state levels of MgP and Pchlide were reduced by 60 and 75%, respectively, compared to WT plants, but *cao* mutants accumulated approximately 1.5–2 fold more porphyrins than the *R211E* lines (Figures 6C,D). The ALA synthesis capacity was similarly reduced in *cao* and *R211E* lines (∼50% of WT level) (Figure 6E). Compared to WT, knockout of *SRP43* or *SRP54* (Figure 7B) resulted in a 50% reduced Chl a and b content (Figure 6B), strongly diminished steady-state levels of MgP and Pchlide (Figures 6C,D) and a 50% reduction in ALA synthesis capacity (Figure 6E). Except for ALA synthesis, deficiency of either factor alone resulted in higher contents of porphyrins and Chl in *chaos* and *ffc* mutants relative to *R211E* lines. In contrast, deficiency of *SRP43* and *54* in the double mutant resulted in *R211E*-like Chl and porphyrin contents (Figures 6B–D) but an even more substantial reduction of the ALA synthesis capacity (Figure 6E). In agreement with previous reports (Wang et al., 2018), GluTR contents were slightly reduced in the *chaos* mutant compared to Ler but accumulated to a higher level in *ffc* than Col-0 WT plants. The *cao* mutant showed a WT-like level of GluTR (Figure 7B).

To elucidate the impact of strongly compromised TPS in *R211E* on the accumulation of Chl-binding proteins, thylakoid protein complexes were solubilized and separated on a gradient blue native (BN-) PA-gel under native conditions and were compared to *gun4-1*, *cao*, *srp43*, and *srp54* single and double mutants (Figure 7). Compared to WT plants, monomeric PSII (PSII-M) and PSI core were enriched, but contents of trimeric LHC (LHC-T) were reduced in the *gun4-1* mutant (Figure 7A). *R211E* lines also accumulated less LHC-T than the WT, but the content was more drastically diminished than in the *gun4-1* knockdown mutant. While PSI-LHCI, PSII dimers (PSII-D) and PSII super complexes (PSII SC) were markedly reduced compared to Col-0, *R211E* also showed a higher level of PSI core and PSII-M. Furthermore, accumulation of the LHCII assembly complex was more pronounced in all genotypes with altered GUN4 levels or function compared to WT thylakoids (Figure 7A). Deficiency of Chl b biosynthesis caused the complete absence of LHCII-T and a substantial accumulation of PSI core and PSII-M in the *cao* mutant. Knockout of SRP54 in *ffc* mutants (Figure 7B) resulted in diminished levels of LHC-T, PSI-LHCI and PSII-D relative to Col-0 (Figure 7A). This effect was even more pronounced in *chaos* mutants (*srp43*), showing a substantial decrease of LHC-T, PSI-LHCI, and PSII-D complexes. Also, strong overaccumulation of the PSI core complex relative to the Ler thylakoids was observed in *chaos*. Intriguingly, however, when both SRP43 and 54 were absent (Figure 7B), the composition of thylakoid protein complexes of *chaos ffc* double mutant resembled that of the *R211E* lines (Figure 7A). Altered accumulation of LHCI and LHCII containing protein complexes in the various genotypes was confirmed by SDS-PAGE analysis using antibodies specific for the LHCA1 and LHCB1 family protein (Figure 7B). The *chaos ffc* double mutant and *R211E* lines accumulated similar, though drastically reduced levels of LHC proteins relative to WT plants. None of the mutants with altered accumulation or function of GUN4 showed a difference in the accumulation of SRP43, 54, or the ALB3 insertase compared to WT plants (Figure 7B). Therefore, the impaired accumulation of Chl-binding proteins in the *R211E* lines is not explained by a differential accumulation of SRP components.

## Discussion

### Effects of GENOMES UNCOUPLED 4 Porphyrin-Binding on Magnesium Chelatase and TPS

In agreement with previous studies on *Synechocystis* and *Arabidopsis* GUN4, mutation of the highly conserved *At*F191 and *At*R211 resulted in the lower affinity of GUN4 toward porphyrins (Figure 1; Verdecia et al., 2005; Adhikari et al., 2009). As shown for *Synechocystis* MgCh and GUN4 (Verdecia et al., 2005), stimulation of rice MgCh is also affected by the GUN4 point mutations confirming the importance of these amino acids for plant GUN4 *in vitro*. Although MgP binding was more severely diminished, binding of the GUN4 point mutants to PIX was also affected. Altered MgP binding to *Syn*GUN4 was previously correlated with reduced MgCh activity (Verdecia et al., 2005), but we cannot conclude from our assays if altered binding to PIX or MgP of GUN4 caused diminished MgCh activity (Figure 1). However, among the different GUN4 versions tested, we found a quantitative difference in the potential to stimulate MgCh. While the R211A protein showed lower affinity toward PIX and MgP compared to F191A, both GUN4 mutants led to similarly reduced MgCh activity (Figure 1). Only for the two R211 substitution mutants, a correlation between porphyrin-binding and MgCh activity was found (Figure 1). In agreement with their general potential to stimulate MgCh *in vitro*, all point mutants at least partially complemented the white phenotype of *GUN4* knockout mutants (Larkin et al., 2003; Peter and Grimm, 2009). Diminished MgCh activity *in vitro* correlated well with the reduced steady-state level of TPS intermediates downstream of the MgCh reaction in the complementation lines (Figure 3), which have also been observed in a *Synechocystis* strain expressing GUN4-W192A mutant with altered porphyrin binding abilities (Kopecna et al., 2015). In the *R211A*#3 line, overexpression of GUN4 (Figures 2, 4) most likely compensated for the perturbed function of GUN4, thereby leading to elevated porphyrin steady-state level compared to the *R211A*#1 line and WT like Chl level and phenotype (Figures 2–4). Despite the overall correlation between the *in vitro* and *in vivo* results, expression of the different GUN4 versions led to phenotypic differences, which cannot solely be explained by altered porphyrin-binding. For instance, the F191A version was less affected in porphyrin-binding compared to R211A (Figure 1), but the expression of *F191A* resulted in a more severe reduction of ALA synthesis, Chl contents (Figure 2), LHC protein abundance (Figure 4) and development of pale leaves (Figure 2) compared to the *R211A*#1 mutant. Considering the equally compromised binding to thylakoid membranes and MgCh (Adhikari et al., 2009, 2011), the differences in the (molecular) phenotypes of *F191A* and *R211A* lines analyzed in the present study are not only explained by lowered porphyrin-binding to GUN4. In addition, *R211A* and *R211E* lines showed entirely different phenotypes despite comparable effects on *in vitro* porphyrin binding (Figure 1). While *R211A* (almost) fully complement the *gun4-2* mutant, even slight overexpression of *R211E* led to only very low porphyrin and Chl level, accumulation of Chl-binding proteins (Figures 4, 7) and strongly impaired growth (Figure 2). Furthermore, expression of *R211E* led to more severe growth retardation and pale green leaves than observed for *gun4-1* (Figure 2), indicating that exchange of R211 to glutamate compromised TPS and Chl accumulation even more substantial than the strong knockdown of GUN4 in *gun4-1*. Interestingly, all genotypes with apparent changes in leaf coloration (i.e., *gun4-1*, *F191A*, and *R211E*) were characterized by significantly reduced ALA synthesis rates (Figure 3), suggesting that diminished ALA biosynthesis contributes to the low TPS intermediate levels and the development of pale green leaves, particularly in *R211E* lines. However, short term ALA feeding in darkness did not restore Pchlide level to WT-like amounts in *R211E* (Figure 5). In agreement with this, a *gun4-2* knock out mutant also showed diminished conversion of the precursor to porphyrins when fed with ALA for 24h in darkness (Peter and Grimm, 2009). Because ALA could partially rescue Chl deficiency of *gun4-2* (Figures 5C,D; Peter and Grimm, 2009), but long-term ALA supply had only a minor impact on Chl accumulation in *R211E* lines (Figure 5), it could be speculated that metabolization of ALA was disturbed in the *R211E* lines. On the other hand, the presence of R211E could also affect the conversion of Pchlide, downstream Chl synthesis or integration into Chl-binding proteins and, thus, would prevent the accumulation of Chl in Chl-binding proteins of the *R211E* lines. Indeed, decreased expression of *PORA* and *B* and reduced accumulation of POR proteins (Figure 4) limits Chl synthesis and certainly contributes to the pale green phenotype of *R211E*. Although major components of the cpSRP pathway for LHC integration into thylakoid membranes accumulated at WT level in *R211E* (Figure 7), we cannot rule out that GUN4 may also affect the accumulation of LHCs downstream of Chl (b) synthesis through post-translational mechanisms involving the interaction with SRP43 as it has been recently reported (Ji et al., 2021). When the integration of LHCs into thylakoid membranes is perturbed, apo-LHC and Chl b cannot be stabilized. However, strong GUN4 knockdown in *gun4-1* and expression of *R211E* led to an increase in the Chl a/b ratio compared to WT plants (Figure 2; Mochizuki et al., 2001) which was even more pronounced compared to mutants impaired in the LHC integration pathway (Figure 6; Wang and Grimm, 2016). This finding suggests that, in particular, the step of Chl b synthesis, catalyzed by CAO, could be compromised in GUN4 deficient or *R211E* expressing mutants. Due to the interdependency of Chl b synthesis and LHC accumulation (Espineda et al., 1999), strongly diminished Chl b content is most likely responsible for the marked decrease of LHC proteins in *gun4-1* and *R211E* (Figures 4, 7). Future analysis will reveal if a mutual connection between GUN4 and the step of Chl b synthesis exists and whether the mutation of R211 affects CAO on the transcriptional or post-translational level.

### GENOMES UNCOUPLED 4:Porphyrin and the Connection With 5-Aminolevulinic Acid Synthesis

5-Aminolevulinic acid synthesis is the rate-limiting step for the biosynthesis of tetrapyrroles, and tight control of ALA formation ensures the biosynthesis of appropriate amounts of TPS intermediates and end products for the integration into Chl-binding proteins (Tanaka et al., 2011; Richter and Grimm, 2019). Several transcriptional and post-translational regulation mechanisms have been reported that tune the activity of enzymes involved in ALA synthesis, among which GluTR is the target of multiple regulatory events (for example, Terry and Kendrick, 1999; Meskauskiene et al., 2001; Goslings et al., 2004; Hou et al., 2018; Richter et al., 2019). Although mechanistic details are unknown, the downregulation of ALA synthesis in mutants of MgCh and downstream enzymes (Papenbrock et al., 2000; Alawady and Grimm, 2005; Wang et al., 2020) might function to prevent the accumulation of TPS intermediates and the formation of ROS. No (direct) physical interaction of GUN4 with enzymes of ALA synthesis has been reported. However, a close connection between GUN4 and ALA synthesis in *Arabidopsis gun4* knockdown mutants (low ALA synthesis capacity) (Figure 3; Peter and Grimm, 2009; Wang et al., 2020) and tobacco *GUN4* overexpression lines (stimulated ALA synthesis) was found (Peter and Grimm, 2009). Also, mutants with impaired regulation of MgCh activity through GUN4 phosphorylation (Richter et al., 2016) or knockout of the GUN4 interacting BALANCE of CHLOROPHYLL METABOLISM1 (BCM1) protein needed for maximum MgCh activity (Wang et al., 2020) showed diminished ALA synthesis rates compared to WT plants. Based on the experimental data published, GUN4 may function in the regulation of ALA synthesis until it is inactivated upon porphyrin accumulation (Peter and Grimm, 2009). Hence, it could be speculated that the function of GUN4 to adjust ALA synthesis depends on its ability to bind porphyrins. However, *F191A* and *R211E* did not accumulate PIX, the substrate of the MgCh reaction, and showed reduced ALA synthesis (Figure 3). Also, *F191A* lines showed significantly reduced ALA synthesis rates despite an R211A like porphyrin binding and stimulation of MgCh *in vitro* (Figure 1). We also found that R211A and R211E mutations impaired porphyrin binding to GUN4 and MgCh activation *in vivo* and *in vitro* (Figures 1, 3), but only *R211E* lines showed significantly reduced ALA synthesis rates (Figure 3). Although we cannot exclude that the minor difference in porphyrin-binding to R211E relative to R211A (Figure 1) might explain the different impact on ALA synthesis, it seems unlikely that the GUN4:porphyrin complex plays a direct role in controlling ALA synthesis. WT-like expression of *HEMA1* and *2* or *GSAT* mRNAs and accumulation of the corresponding enzymes (Figure 4) in the different mutants indicated that impaired porphyrin-binding to GUN4 did not adjust the expression of ALA synthesis enzymes. Therefore, downregulation of ALA synthesis was mediated through a post-translational mechanism independent of GUN4’s porphyrin-binding abilities (Figure 4). Because ALA synthesis was significantly reduced in all genotypes with a strongly altered accumulation of Chl-binding proteins and photosynthetic complexes (Figure 7), diminished ALA synthesis could also be explained by reduced photosynthetic activity or accumulation/function of photosynthetic complexes. For example, strong knockdown of GUN4 in *gun4-1* and expression of *F191A* and *R211E* led to impaired Chl b accumulation (relative to Chl a), and ALA synthesis in Chl b deficient *cao* mutant resembled that of *R211E* (Figure 6). Therefore, a mutual connection between CAO activity and GUN4 could exist, and diminished ALA synthesis would be a consequence of altered Chl b biosynthesis or the reduced accumulation of Chl-binding proteins. On the other hand, the lack of SRP43 and 54 also led to markedly decreased ALA synthesis capacity (Figure 7; Wang et al., 2018), and *R211E* lines resembled the *chaos ffc* double mutant. Given the physical interaction of SRP43 with GluTR (Wang et al., 2018) and GUN4 (Ji et al., 2021) and diminished GUN4 contents and ALA synthesis in *srp43* mutant (Figure 7; Ji et al., 2021), it would be of future interest to test whether the interaction of GUN4 with SRP components is modified by F191A or R211E mutations thereby leading to reduced ALA synthesis (Figure 7). In addition, knockout of BCM1, which also interacts with GluTR, resulted in lower ALA synthesis capacity compared to WT plants, and ALA formation depends on both GUN4 and BCM1 (Wang et al., 2020). Hence, it could be speculated that F191A and R211E mutations affect the interaction of GUN4 with BCM1 resulting in reduced ALA synthesis capacity in *F191A* and *R211E*. Future analysis of GUN4 point mutants will reveal how these amino acids affect the interaction with the different partners and how these interactions are connected to the regulation of ALA synthesis. To this end, direct interaction analysis using microscale thermophoresis or pull-down assays with recombinant proteins and CoIP experiments with plant extracts could be performed. Furthermore, interaction assays between MgCh subunits and the different GUN4 variants in the presence and absence of porphyrins will show how the mutation of conserved amino acids – particularly R211E – influences GUN4’s potential to interact with MgCh subunits.

### Altered Porphyrin-Binding Has No Impact on GENOMES UNCOUPLED 4 and TPS Enzyme Stability

Previous work on *GUN4* deficient *Arabidopsis* mutants revealed a critical role of GUN4 during growth in light/dark cycles (Larkin et al., 2003; Peter and Grimm, 2009). The accumulation of unbound porphyrin intermediates is a significant threat to plants (for example, Meskauskiene et al., 2001; Peter et al., 2010) because excited porphyrins can return to their ground state by energy transfer to molecular oxygen, thereby promoting the formation of singlet oxygen. The highly reactive singlet oxygen interferes with the function of various biomolecules (Kim et al., 2008) and can even stimulate the genetically encoded programmed cell death (Meskauskiene et al., 2001; Kim and Apel, 2013). Previously, it was hypothesized that GUN4 might function as a buffer for excessive porphyrins, and efficient shielding of porphyrins from the interaction with molecular oxygen could diminish the phototoxic effects arising from unbound porphyrin accumulation (Peter and Grimm, 2009; Adhikari et al., 2011; Li et al., 2021). This hypothesis is further supported by GUN4’s ability to bind various TPS pathway intermediates (Adhikari et al., 2009; Hu et al., 2021; Zhang et al., 2021). The modification or degradation of the GUN4:porphyrin complex was proposed as an efficient way to catabolize a surplus of TPS intermediates. Also, lowered content or degradation products of GUN4 could function as a signal for the downregulation of ALA synthesis to avoid further accumulation of porphyrins (Peter and Grimm, 2009) or to permit the adjustment of nuclear gene expression by plastid-to-nucleus communication pathways (Brzezowski et al., 2014). These proposed functions of the GUN4:porphyrin complex imply that GUN4 is degraded upon porphyrin binding in light. However, based on the *in vivo* analysis of porphyrin-binding mutants of GUN4 (Figures 1, 2), no correlation between the stability and GUN4’s porphyrin-binding abilities was found. In all the analyzed genotypes, the expression of the transgenes (Figure 2C) correlated with the accumulation of GUN4 protein (Figure 4A). This finding is in agreement with stable GUN4 contents after the accumulation of high amounts of porphyrins induced by ALA feeding of *Arabidopsis* WT leaves (Richter et al., 2019). However, Adhikari et al. (2011) reported that porphyrin-binding deficient F191A and R211A expressed in *gun4-2* were degraded while the WT GUN4 remained stable upon high light (HL) shift. Because the GUN4-1 mutant protein with increased affinity for porphyrins (Davison et al., 2005) was similarly degraded in HL treated *gun4-1* mutants (Adhikari et al., 2011), GUN4 degradation upon HL shift is most likely not connected to the formation of GUN4:porphyrin complexes. More recently, a stabilizing effect of GUN4 on the MgCh H1 subunit in the presence of PIX, biliverdin (BV) and other linear tetrapyrroles was reported in *Chlamydomonas reinhardtii* (Hu et al., 2021; Zhang et al., 2021). Also, for *Synechocystis*, a connection between porphyrin binding to GUN4 and accumulation of CHLH after recovery from nitrogen depletion has been reported (Kopecna et al., 2015). In contrast to unicellular organisms, *Arabidopsis gun4* mutants accumulated at least WT like CHLH amounts (Figure 4A) and CHLH is not destabilized in, for example, *R211E* lines expressing GUN4 with strongly impaired porphyrin binding (Figure 4A). Although bilin binding to *At*GUN4 was reported and binding to *Synechocystis* GUN4 depends on the conserved amino acids involved in binding of porphyrin intermediates (Hu et al., 2021), we propose that the GUN4:porphyrin complexes do not determine the stability of MgCh subunits in higher plants. This assumption is further supported by previous analysis of *gun4-2* expressing either *F191A* or *R211A* (Adhikari et al., 2011). After shifting plants from growth light to high light intensities, the abundance of the GUN4 variants with altered porphyrin binding decreased, but CHLH contents remained stable (Adhikari et al., 2011). Furthermore, knockout of *GUN4* led to post-translational destabilization of MgPMT (*CHLM*) and POR, indicating a function of GUN4 on enzymes downstream of MgCh (Peter and Grimm, 2009). Contents of MgPMT and POR were also reduced in the *R211E* lines but accumulated to WT-like level in *R211A* or *F191A* lines (Figure 4A). Hence, diminished contents of these two enzymes cannot be solely explained by altered porphyrin-binding to GUN4. Instead, the reduced expression of *PORA* and *B* in *R211E* (Figure 4A) explained the lower contents of POR. However, it remained open if the changed *POR* expression is a direct consequence of perturbed GUN4 function or strongly impaired Chl biosynthesis and its consequences in *R211E* lines (e.g., accumulation of Chl-binding proteins) (Figures 6, 7).

## Data Availability Statement

The original contributions presented in the study are included in the article/Supplementary Material, further inquiries can be directed to the corresponding author.

## Author Contributions

ASR conceived and supervised the experiments and wrote the article. VF, CG, and ASR performed the experiments. All authors contributed to manuscript revision, read, and approved the submitted version.

## Conflict of Interest

The authors declare that the research was conducted in the absence of any commercial or financial relationships that could be construed as a potential conflict of interest.

## Publisher’s Note

All claims expressed in this article are solely those of the authors and do not necessarily represent those of their affiliated organizations, or those of the publisher, the editors and the reviewers. Any product that may be evaluated in this article, or claim that may be made by its manufacturer, is not guaranteed or endorsed by the publisher.
