# Supplementary material for: Impact of Porphyrin Binding to GENOMES UNCOUPLED 4 on Tetrapyrrole Biosynthesis in planta
Source: Front Plant Sci. 2022 Mar 15;13:850504. doi: 10.3389/fpls.2022.850504 (PMC8967248; doi:10.3389/fpls.2022.850504)
Supplement: Supplementary file 1 [file Table_1.docx]

**Supplementary Figure**


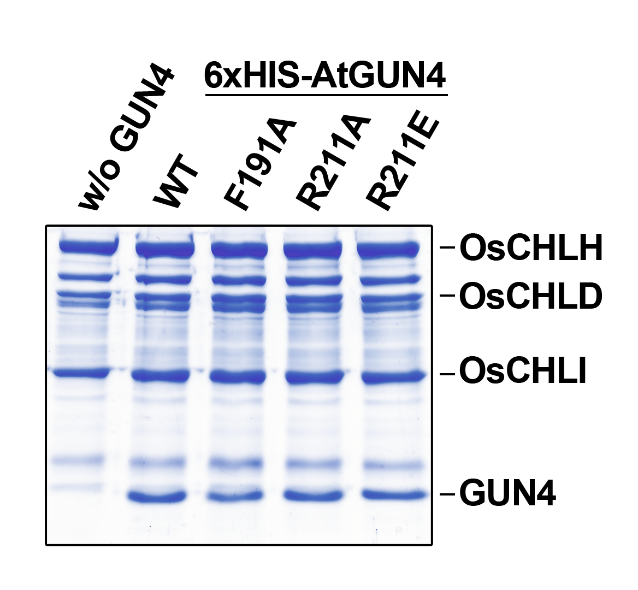


**Figure S1 – Confirmation of the presence of recombinant GUN4 and MgCh subunits in the MgCh assay.**

Aliquots of the MgCh reaction mixture were mixed with loading buffer after stop of the assay and separated on SDS-PAGE followed by coomassie blue staining. Os, Oryza sativa; At, Arabidopsis thaliana.
